# A species of *Coprococcus* is related to BMI in patients who underwent malabsorptive bariatric surgery and its abundance is modified by magnesium and thiamin intake

**DOI:** 10.3389/fendo.2025.1613221

**Published:** 2025-11-06

**Authors:** Fernando Suárez-Sánchez, Evelyn Pérez-Ruiz, Claudia Ivonne Ramírez-Silva, Mario Antonio Molina-Ayala, Sandra Rivera-Gutiérrez, Lizbel León-Solís, Lázaro García-Morales, Arturo Rodríguez-González, César Martínez-Ortiz, Luis Axiel Meneses-Tapia, Miguel Cruz-López

**Affiliations:** 1 Medical and Biochemistry Research Unit, Specialty Hospital, Centro Médico Nacional Siglo XXI, Instituto Mexicano del Seguro Social, Mexico City, Mexico; 2 Faculty of Chemistry, Doctorate Program in Medical, Dental and Health Sciences, Clinical and Experimental Health Research, Universidad Nacional Autonoma de México, Mexico City, Mexico; 3 Department of Maternal, Child, and Adolescent Nutrition. Center for Nutrition and Health Research. National Institute of Public Health. Cuernavaca, Morelos, Mexico; 4 Diabetes and Obesity Clinic, Specialty Hospital, Centro Médico Nacional Siglo XXI, Instituto Mexicano del Seguro Social, Mexico City, Mexico; 5 Department of Microbiology, Escuela Nacional de Ciencias Biológicas, Instituto Politécnico Nacional, Mexico City, Mexico; 6 Department of Molecular Biomedicine, Centro de Investigación y de Estudios Avanzados del Instituto Politécnico Nacional, Mexico City, Mexico

**Keywords:** magnesium, thiamin, *Coprococcus*, bariatric surgery, acetate, microbiota

## Abstract

**Background:**

Severe obesity is associated with metabolic alterations and an increased risk of developing type 2 diabetes. Bariatric surgery, especially malabsorptive procedures, results in significant clinical improvements and induces changes in the gut microbiota composition. This study aimed to identify bacterial taxa associated with changes in body mass index (BMI) in patients undergoing bariatric surgery and to explore their relationship with nutrient intake.

**Methods:**

Individuals with severe obesity were recruited prior to and following bariatric surgery. Fecal DNA was extracted and the V4 region of the 16S rRNA gene was sequenced. Quality control and taxonomic classification were performed using QIIME2 and the Greengenes database. Nutrient intake was assessed through a 7-day dietary recall. Anthropometric measurements and blood samples were collected to evaluate clinical variables. Statistical analyses were conducted using R software.

**Results:**

Significant differences in gut microbiota diversity were observed post-bariatric surgery. The Shannon and Simpson diversity indices decreased significantly after surgery (p < 0.001). Beta diversity analysis (Bray-Curtis, Weighted and Unweighted UniFrac) also showed significant differences between pre- and post-surgery samples (p = 0.001). The abundance of a species within the genus *Coprococcus* was positively correlated with magnesium and thiamin intake in post-surgery patients (rho = 0.816, p_FDR_ = 0.029 and rho = 0.812, p_FDR_ = 0.029, respectively). Furthermore, *Coprococcus* sp. abundance was positive associated with BMI in pre-surgery individuals (p = 0.043) but negative associated with BMI in post-surgery individuals (p = 0.036). Several taxa within the order *Clostridiales* and microbial metabolic pathways involved in sugar degradation, acetate, thiamin (vitamin B1) and some amino acid production were enriched prior to surgery.

**Conclusions:**

The abundance of a species of the genus *Coprococcus* showed an inverse relationship with BMI in pre-surgery and post-surgery individuals and correlated positively with magnesium and thiamin intake in patients who underwent a malabsorptive bariatric surgery. These findings suggest that optimizing micronutrient intake may enhance the beneficial effects of bariatric surgery on BMI by favorably modulating gut microbiota composition.

## 1 Introduction

Obesity is a disease characterized by the excessive accumulation of fat at several body sites. Fat deposits around the waist are particularly detrimental and are associated with the onset of other pathologies such as cardiovascular disease and type 2 diabetes (T2D) (1). A body mass index (BMI) of 40 or higher is classified as severe obesity (2). In addition to the metabolic alterations inherent to obesity, patients often experience mobility problems and everyday life is severely affected. In most cases, dietary and exercise interventions have limited impact on weight loss; therefore, other approaches, such as bariatric surgery, are employed (3). Although different types of bariatric surgery exist, the use of malabsorptive bariatric surgery has demonstrated particularly favorable outcomes in obese patients. The most common procedure is Roux-en-Y gastric bypass (RYGB) although one-anastomosis gastric bypass (OAGB) has also been used successfully. Both surgeries involve rearranging the intestines in a way that limits nutrients absorption by bypassing the proximal portion of the small intestine and facilitating the passage of poorly digested food to the distal small intestine and colon (1, 4). These changes contribute to modifications in gut bacterial diversity and balance. This new microbial equilibrium influences the capacity of the gut bacteria to perform biologically relevant functions, including the synthesis of essential vitamins, the production of signaling molecules and digestion of nutrients (5).

Prebiotics and probiotics have been utilized to modulate the abundance of gut bacteria in the host. For example, fiber intake has been widely recommended to promote the growth of short-chain fatty acid (SCFA)-producing bacteria (6). However, the benefits of specific bacteria should be evaluated in a context-dependent manner since some bacteria such as the SCFA-producing bacteria *Coprococcus* has been linked to both positive and negative traits (7–16). Although less is known about the role of other dietary micronutrients in shaping the gut flora, evidence suggests that vitamins, minerals and trace elements in the diet can alter gut bacteria composition leading to improvements of clinical variables (17, 18). Particularly, thiamin and magnesium have been linked to changes in the abundance of *Bacteroides*, *Alistipes*, *Bacilli* and *Bifidobacterium* as well as to improvements in blood glucose, medium-chain fatty acids and some metabolic syndrome parameters (17, 19, 20).

In this study, we identified a species of *Coprococcus* whose abundance was higher in patients with greater BMI after bariatric surgery. The response of *Coprococcus* sp. to dietary intake was subsequently investigated, and we identified two micronutrients (magnesium and thiamin) that correlated with its abundance in the gut. Given the essential role of micronutrients in microbiota composition and the altered nutrient absorption following bariatric surgery, understanding the nutrients-microbiota relationship is critical for post-operative dietary guidance. Changes in several other bacterial taxa and associated metabolic pathways were observed in patients who underwent malabsorptive bariatric surgery.

## 2 Material and methods

### 2.1 Study population

Participants were recruited at the Specialty Hospital, National Medical Center Century XXI, Mexican Social Security Institute. A total of 21 individuals, all classified as severely obese, were scheduled to undergo an elective malabsorptive bariatric surgery (Roux-en-Y gastric bypass [RYGB] or one-anastomosis gastric bypass [OAGB]). Patients were excluded if they had undergone a previous cholecystectomy or bariatric surgery at the time of the recruitment. Other exclusion criteria, either at recruitment or after surgery were having taken antibiotics within the previous three months or presenting signs of gastrointestinal infection. All patients signed an informed consent form prior to the collection of any blood and fecal samples, anthropometric measurements, or dietary intake interviews. This study was approved by the National Ethics Committee of the Mexican Social Security Institute and was conducted in accordance with the ethical guidelines of the Declaration of Helsinki.

### 2.2 Sample collection and diet

Blood samples were collected before and 109 ± 28 days after patients underwent a bariatric surgery. Patients were instructed to fast for 10 hrs. prior to sampling and glucose, triglycerides, total cholesterol, HDL, LDL and insulin concentrations were measured in serum. Fecal samples were received, aliquoted and stored at -70°C by the laboratory personnel the same days that the blood samples were drawn. Dietary intake during the previous seven days was assessed using a Semi-Quantitative Food Frequency Questionnaire (SFFQ). This tool was based on the National Health and Nutrition Survey SFFQ and adapted to include foods available in different seasons, such as fruits and vegetables, reflecting seasonal variation in food consumption (21, 22). The questionnaire included 191 food items divided into 16 sections.

### 2.3 Micro- and macronutrient estimations

Energy and nutrients intake were calculated using the methodology applied in the National Health and Nutrition Survey of Mexico (ENSANUT) (23). The nutrients database was built using items from the Mexican Food Database (MFD; BAM, acronym in Spanish: *Base de datos de los Alimentos Mexicanos*) version 1.1 which contains information on 1978 foods, recipes, and beverages (24). To improve the accuracy of the dietary intake estimation, nutrient retention factor (NRF) were applied according to the recommendation of the United States Department of Agriculture (USDA) and Bognar recommendations (25, 26). Data processing was performed in Stata v.14.0 (Stata Corporation).

### 2.4 DNA extraction and 16S rDNA sequencing

Fecal samples were collected by patients within 24 hrs. prior to delivery to laboratory personnel. Samples were aliquoted and stored at -80°C. DNA was extracted from 150–200 mg of stool using the QIAamp DNA Mini Kit (Qiagen) following the manufacturer's instructions, with the addition of a mechanical lysis step at the beginning of the extraction procedure. The mechanical lysis was performed with the TissueLyser LT equipment (Qiagen) for 5 minutes at 60 cycles/second. DNA integrity was assessed by 2% agarose gel electrophoresis and purity was determined by the 260/280 ratio using a BioTek Epoch™ Microplate Spectrophotometer (Agilent Technologies).

Library preparation and sequencing of the V4 hypervariable region of the bacterial 16S rDNA gene were performed following the methodology described by Kozich Westcott (27). Briefly, 20 ng of DNA, quantified with Qubit™ (Thermo Fisher Scientific) and the Quant IT™ dsDNA HS Assay Kit were used as a template for the amplification of the V4 region (25 amplification cycles) using the AccuPrime™ Pfx SuperMix high-fidelity DNA polymerase from Invitrogen™ (Thermo Fisher Scientific). Amplicons were purified with Beckman Coulter™ AMPure XP 1.8X beads (Life Sciences) and fragment size was confirmed using the 4200 TapeStation High Sensitivity DNA Reagent Kit (Agilent Technologies). DNA concentration was re-quantified with a Qubit™ instrument and the Quant IT™ dsDNA HS Assay Kit (Thermo Fisher Scientific) and the concentration was adjusted to 4 nM. Equimolar pooling was followed by a new quantification using the Qubit™ and the Quant IT™ dsDNA HS assay kit (Thermo Fisher Scientific) and denaturation with NaOH. Then, the DNA pooling was spiked with PhiX (PhiX Control Kit v3 Illumina). Paired-end DNA sequencing was performed on a MiSeq system (Illumina) with the MiSeq Reagent Kit V3 (600-cycle) (Illumina).

### 2.5 Data analysis

Sequencing quality control and identification of the amplicon sequence variants (ASVs) were performed with QIIME2 and the DADA2 package (28, 29). Features with less than 11 counts across all samples as well as features that appeared in only one sample were removed. Rarefaction with a sampling depth of 21,348 was performed. The rarefied table was later transformed to relative frequencies. Alpha and beta diversity were calculated in QIIME2 and the taxonomic assignation was performed using the Greengenes database (30). Statistical distribution of the variables was evaluated with the Shapiro–Wilk test. Phenotypic variables and alpha diversity were compared using paired Student’s t test or paired Wilcoxon signed ranked test depending on the type of statistical distribution. The abundance of one species of the genera *Coprococcus* (taxonomic assignation by QIIME2 was g_*Coprococcus*;s_ but hereafter referred as *Coprococcus* sp.) was stratified as “higher than” or “lower than” the median abundance in pre- and postsurgery groups. BMI was compared between these stratified groups using the Wilcoxon signed ranked test. PERMANOVA was employed to assess differences in Bray–Curtis, weighted and unweighted UniFrac data.

Pre- and post-surgery bacterial abundance differences were assessed with the paired Wilcoxon signed rank test. To account for multiple testing, p-values were adjusted using False Discovery Rate (FDR). Similarly, to control for false positives, FDR-adjusted p-values were calculated in Spearman correlations analysis of bacterial abundance with energy and nutrients intakes (macro- and micronutrients).

Functional inference was performed using Picrust2 to identify enriched metabolic pathways at the species level (31). Effect size was computed using the *aldex* function from the ALDEx2 package in R (version 4.3.0 [2023-04–21 ucrt]). All statistical analysis and plots were generated in R.

## 3 Results

### 3.1 Phenotypic characterization

Malabsorptive bariatric surgery improved several anthropometric and biochemical parameters. On average, patients lost 28 kg, which represents approximately 24% of their initial body weight. Most patients were reclassified as having class I obesity (30 ≤ BMI < 35) instead of severely obesity (BMI ≥ 40, their initial classification). Glucose, triglycerides, LDL and total cholesterol serum levels decreased after the surgery (**Table 1**).

**Table 1:** Anthropometric and biochemical characteristics of the population.

	All groups n = 42	Presurgery n = 21	Postsurgery n = 21	p value
Age (years)	44.7 ± 7.0	44.7 ± 7.1	44.7 ± 7.1	1
Gender (female)	26 (62%)	13 (62%)	13 (62%)	0.38
Weight (kg)*	106.1 [86.2 - 130.4]	117.0 [102.0 - 139.5]	88.9 [77.8 - 116.7]	**0.049**
BMI*	38.8 [33.7 - 46.6]	42.6 [39.4 - 47.2]	33.7 [30.1 - 37.0]	**0.0071**
Waist (cm)	126.4 ± 24.6	132.5 ± 24.0	119.6 ± 24.3	0.28
Hip (cm)	134.4 ± 20.5	139.0 ± 19.9	129.2 ± 20.5	0.31
WHR	0.94 ± 0.11	0.95 ± 0.09	0.93 ± 0.13	0.63
Glucose (mg/dL)*	84.4 [79.3 - 90.8]	87.8 [84.7 - 93.7]	80.0 [78.9 - 84.3]	**0.004**
Triglycerides (mg/dL)*	103.4 [88.1 - 146.9]	134.6 [96.1 - 168.2]	95.8 [87.6 - 118.3]	**0.019**
HDL (mg/dL)	43.4 ± 12.4	46.1 ± 10.1	40.6 ± 14.1	0.14
LDL (mg/dL)*	102.0 [77.3 - 122.0]	112.3 [97.6 - 143.0]	88.6 [67.3 - 103.5]	**0.008**
Cholesterol (mg/dL)	166.6 ± 46.8	187.2 ± 42.3	146.0 ± 42.5	**0.0016**

* The Wilcoxon signed rank test was applied when the variable showed a non-normal statistical distribution; otherwise, a paired Student’s t-test was used. The McNemar test was applied to compare sex distributions. p-values < 0.05 are shown in bold. BMI, Body mass index; WHR, Waist-to-hip ratio; HDL, High-density lipoprotein; LDL, Low-density lipoprotein.

### 3.2 Alpha and beta diversity

**Figure 1:**
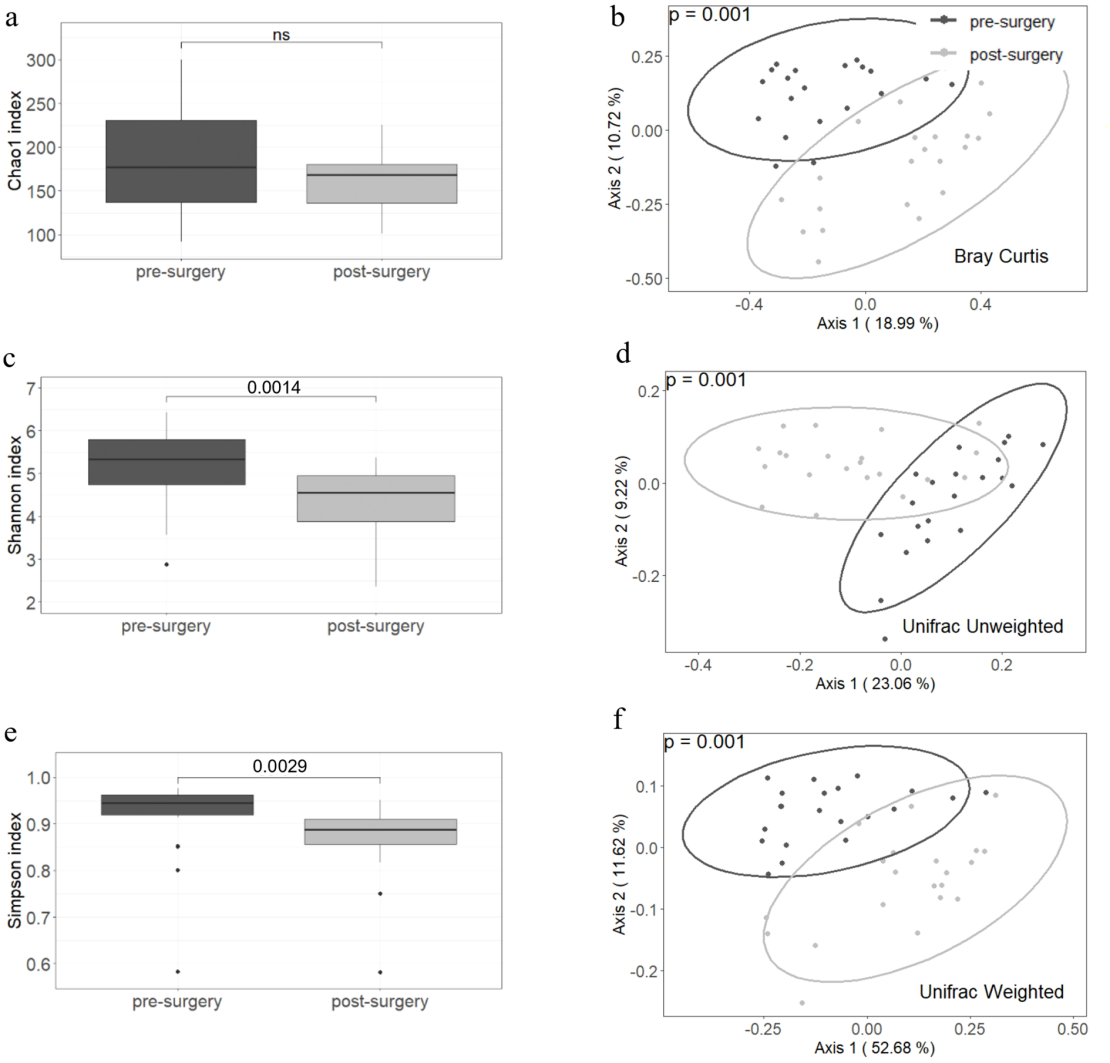
Alpha and beta diversity are different before and after a malabsorptive bariatric surgery. The Chao1 index **(a)** did not differ between groups but Shannon and Simpson indices **(c, e)** decreased after surgery. Beta diversity assessed by Bray-Curtis, Unweighted and Weighted Unifrac showed significant differences between pre- and post- surgery samples **(b, d, f)**. Twenty-one individuals were included in each group. The paired Wilcoxon signed ranked-test was used to compare the alpha diversity between groups and PERMANOVA to assess statistical differences in beta diversity.

Microbiota richness assessed by the Chao1 index did not differ significantly when comparing samples collected before and after surgery (**Figure 1a**). However, the Shannon and Simpson indices showed significant differences between the two groups (p values of 0.0014 and 0.0029, respectively), with higher diversity index observed in the pre-surgery group (**Figures 1c, e**). Beta diversity was different for Bray–Curtis, weighted and unweighted UniFrac indices between the compared groups. In all three cases, the PERMANOVA p value was 0.001 (**Figures 1b, d, f**).

### 3.3 Correlation between microbiota and nutrients intake

**Figure 2:**
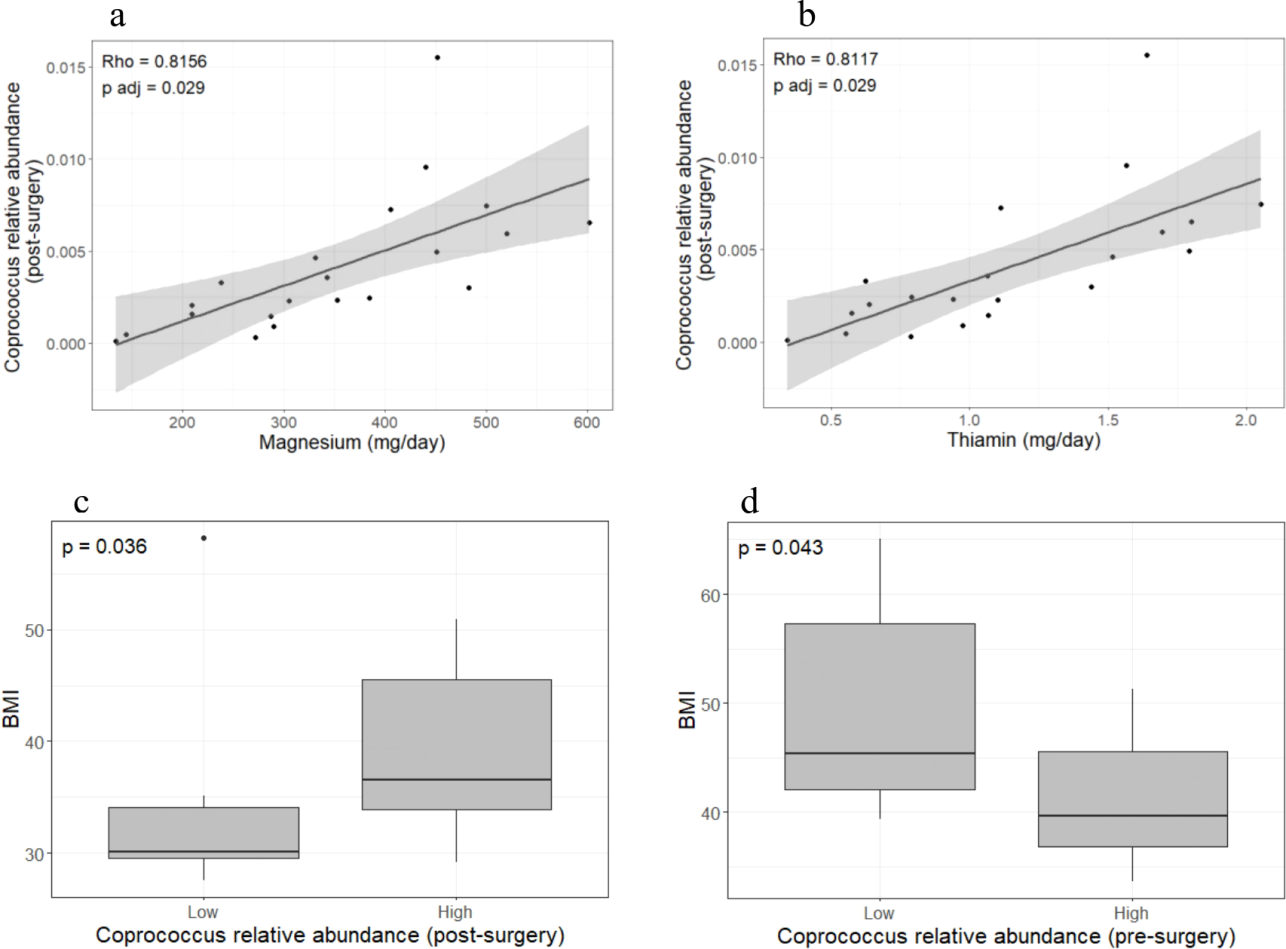
*Coprococcus* sp. abundance is related to micronutrients intake and BMI. Magnesium and thiamin intake was positively correlated with *Coprococcus* sp. abundance in the post-surgery group **(a, b)** but not in the pre-surgery group (data not shown). Patients with low *Coprococcus* sp. abundance (dichotomized into lower and higher quantiles) showed lower BMI in the post-surgery group **(c)** but the opposite was observed in the pre-surgery group **(d)**. Twenty-one individuals were included in each group. Spearman correlation was used to assess the relationship between bacteria abundance and dietary intake. Due to the large number of bacteria and micronutrients analyzed, p-value were adjusted by FDR. Statistical differences in BMI between low- and high- abundance *Coprococcus* sp. groups were calculated with the Wilcoxon signed ranked-test. Analyses and plots were performed in R (version 4.3.0 [2023-04–21 ucrt]).

Carbohydrates, proteins, and total energy intake showed a trend toward decreasing in the post-surgery group; however, the differences did not reach statistical significance. Magnesium and thiamin intake was positively correlated with the relative abundance of *Coprococcus* sp. in the post-surgery group (rho = 0.82, p_FDR_ = 0.029; rho = 0.81, p_FDR_ = 0.029, respectively) (**Figures 2a, b**) but not in obese individuals (data not shown). Further comparison of anthropometric and biochemical parameters between the low (≤ median) and high (> median) *Coprococcus* sp. abundance groups showed that BMI was greater in post-surgery individuals with higher *Coprococcus* sp. relative abundance (p = 0.036; **Figure 2c**). This relationship was reversed in the pre-surgery group where BMI decreased as *Coprococcus* sp. relative abundance increased (p = 0.043, **Figure 2d**). When the energy intake was compared by *Coprococcus* sp. abundance level, post-surgery patients in the higher-abundance group had greater energy intake (2066.4 kcal [1497.9 kcal – 2262.8 kcal] vs. 1379.6 kcal [1007.8 kcal – 1680.1 kcal], p = 0.024). No differences in energy intake was observed in the pre-surgery group.

### 3.4 Changes in the microbiota before and after a bariatric surgery

**Figure 3:**
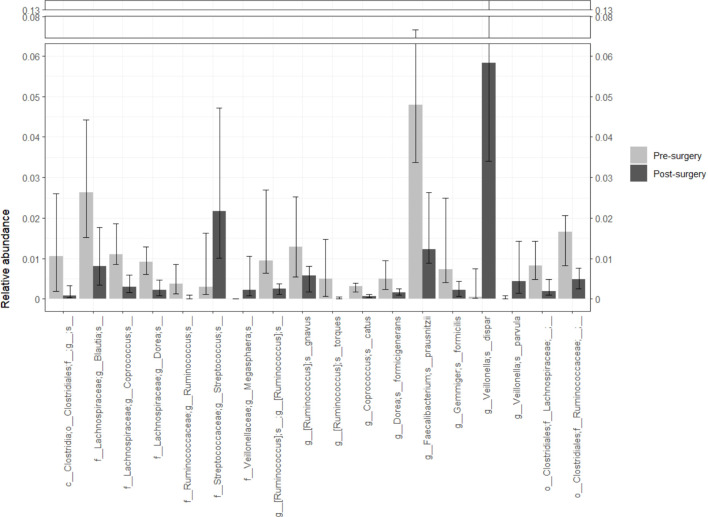
Bacterial abundance changes after patients underwent a malabsorptive bariatric surgery. Only bacteria with p_FDR_ < 0.05 and a median sequence count of 50 in the rarefied abundance table are shown although the analysis included all bacteria identified by sequencing of the V4 variable region of the rDNA gene. A reduction in the abundance of most bacteria was observed. The genera *Streptococcus*, *Megasphaera* and the species *Veillonella dispar* and *Veillonella parvula* were more abundant in the post-surgery group. Statistical differences in bacterial abundance between groups were assessed using the paired Wilcoxon signed ranked-test. The p values were adjusted by FDR to control for false positives. Twenty-one individuals were included in each group. Analyses and plots were generated with R (version 4.3.0 [2023-04–21 ucrt]).

Eighteen bacterial species (meeting the criteria of p_FDR_ ≤ 0.05 and a median sequences count of 50 in the rarefied abundance data across the studied population) exhibited different relative abundances before and after a bariatric surgery (**Figure 3**). Most bacteria decreased in abundance after surgery (all belonging to the order *Clostridiales*). Only the abundance of *Streptococcus*, *Megasphaera*, *Veillonella dispar* and *Veillonella parvula* increased post-surgery. Bacteria that differed between groups belonged to the order *Clostridiales* and were clustered in three families: *Lachnospiraceae*, *Ruminococcaceae* and *Veillonellaceae*. At the phylum level, *Bacteroidetes* and *Fusobacteria* increased after surgery (0.32 [0.17-0.43] vs. 0.52 [0.42-0.60], p_FDR_ = 0.014 and 0.0 [0.0-0.0] vs. 0.0 [0.0-0.02], p_FDR_ = 0.002; respectively), whereas *Firmicutes* decreased (0.61 [0.49-0.73] vs. 0.38 [0.30-0.48], p_FDR_ = 0.002) (**Supplementary Data S1**).

### 3.5 Enriched bacterial metabolic pathways

**Figure 4:**
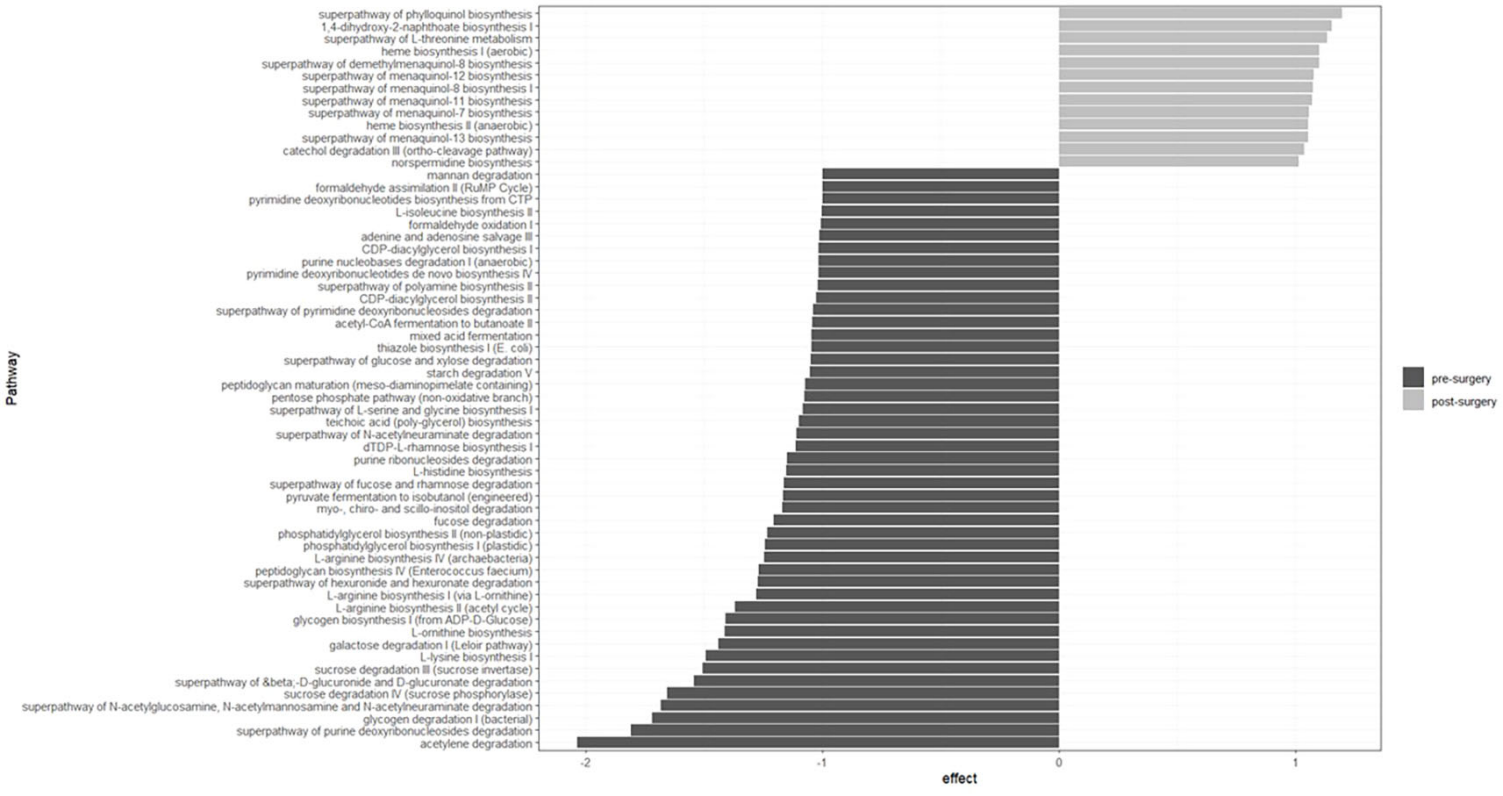
Bacterial metabolic pathways enriched in the pre- and post-surgery groups are related to amino acid, thiamin and K biosynthesis, sugar degradation and acetate production. Twenty-one individuals were included in each group. Metabolic pathways were predicted with Picrust2 and the effect size estimated using the ALDEx2 package in R (version 4.3.0 [2023-04–21 ucrt]).

The enrichment of bacterial metabolic pathways is shown in **Figure 4**. In the pre-surgery group, the main enriched pathways were related to amino acid (lysine, ornithine, arginine, histidine, serine, glycine and isoleucine) and thiamin biosynthesis, sugars (sucrose, galactose, fucose, rhamnose, glucose, xylose and mannan) and starch degradation, acetate production and glycogen biosynthesis and degradation. In contrast, in the post-surgery group, pathways related to vitamin K synthesis were enriched.

## 4 Discussion

Bariatric surgery is a procedure that has been shown to substantially reduce weight and improve metabolic alterations associated with diabetes. Major remodeling of the digestive tract contributes to these changes by increasing the release of anorexigenic hormones and decreasing nutrient absorption but also by altering the gut microbiota composition (32). Indeed, our study revealed that bacterial diversity (alpha and beta) varied greatly after bariatric surgery with several species decreasing in abundance. The abundance of one of these bacteria (g_*Coprococcus*;s_ according to QIIME2 taxonomic assignation but referred as *Coprococcus* sp.) showed an opposite relationship with BMI before and after surgery and was positively correlated with magnesium and thiamin intake in the post-surgery group.

The results of this study are consistent with previously published findings showing that bariatric surgery improves patients´ health. Patients in the present study lost an average of 24% of their initial weight and they were reclassified from class III obesity to class I obesity. At baseline, 71.4% of the patients were taking some type of hypoglycemic medication, but this proportion was reduced to 14.3% after surgery. Biochemical variables such as fasting glucose, triglycerides, LDL and total cholesterol improved as well (**Table 1**).

Improvement in anthropometric and biochemical parameters was accompanied by a decrease in Shannon and Simpson diversity indices (**Figure 1**). This reduction in diversity is attributed to the extensive remodeling of the gut, which prevents the transit of food through the first portion of the small intestine, leading to incomplete digestion and overall changes in the gut microenvironment. Beta diversity estimated by Bray–Curtis, unweighted and weighted UniFrac indices showed clear differences between the compared groups (**Figure 1**). These results indicate that the bacterial composition is strongly modified after malabsorptive bariatric surgery. At the phylum level, the abundance of *Firmicutes* decreased, while that of *Bacteroidetes* and *Fusobacteria* increased (**Supplementary Material S1**). These findings are consistent with reports in the literature indicating that the microbiota of obese patients is enriched in *Firmicutes* (33). At lower taxonomic levels and after adjusting the p value by false discovery rate, we identified 18 taxa that varied in abundance (**Figure 3**). Most taxa decreased in abundance after bariatric surgery (14 out of 18). Taxa that decreased in abundance belonged to the order *Clostridiales* and the families *Lachnospiraceae* and *Ruminococcaceae*. The genomes of members of both families are known to contain ATP-binding cassette (ABC) genes and other genes encoding enzymes that participate starch and sugars degradation. Hydrolysis of these sugars results in the production of SCFAs (butyrate, acetate, and propionate) (34). Although increased SCFA production is generally considered beneficial for gut homeostasis, when combined with a greater bacterial capacity to extract energy from the diet, it may contribute to the obese phenotype. In line with this interpretation, we identified enrichment of several pathways involved in starch and sugar degradation (i.e. xylose, galactose, N-acetylmannosamine, N-acetylneuraminate, N-acetylglucosamine, mannan, glucose, rhamnose, fucose, and sucrose) in obese patients (**Figure 4**).

Another pathway that was enriched in the pre-surgery group was related to the acetylene degradation. The end product of this pathway is microbiota-derived acetate. Evidence indicating that acetate production by the microbiota due to high fructose consumption (which is relatively common in obese patients) leads to activation of hepatic ACSS2 enzyme which transforms acetate to lipogenic acetyl-CoA (35). This occurs in addition to the conversion of glucose and fructose to fatty acids in the liver (36). Thus, increased acetate and lipogenic acetyl-CoA production combined with high-caloric diets in obese pre-surgery patients may contribute to the maintenance of the obese phenotype.

Microbial glycogen degradation and glycogen biosynthesis pathways were enriched in the pre-surgery group as well. The enrichment of both energy-related pathways indicates that the microbiota of severely obese patients has the capacity to manage the energy surplus obtained due to the patients’ intake of high-caloric diets.

Previous reports indicate that approximately 27% of patients undergoing bariatric surgery have thiamin deficiency (37). This is clinically relevant because these patients require lifelong vitamin B1 (thiamin) supplementation. Thiamin is crucial and indirectly involved in the electron transport chain and extraction of energy from carbohydrates but also is required for bacterial growth (38). This essential vitamin is obtained from the diet and as a product of bacterial metabolism. However, not all bacteria have the genes necessary for its *de novo* synthesis (39). In this study, we found that the abundance of three bacteria (*Faecalibacterium prausnitzii*, *Gemmigers formicilis* and *Ruminococcus*) that require thiamine for their survival but cannot produce thiamine by themselves decreased (**Figure 3**). These bacteria belong to the *Ruminococcaceae* family (39). This abundance decrease corresponds to a reduction of the thiazole biosynthesis pathway after the surgery (**Figure 4**).

Although thiamin is required for normal physiological processes, our results suggest that fine tuning of thiamin intake is desirable in post-surgery patients. We observed that thiamin intake was positively correlated with *Coprococcus* sp. abundance which in turn was related to higher BMI after surgery (**Figures 2b, c**). We further observed that the *Coprococcus* sp. relative abundance in the post-surgery group was positively correlated with magnesium intake (**Figure 2a**) which is required for the proper activation of vitamin B1 (20, 40). The above-mentioned evidence suggest that thiamin and magnesium intake must be such that it does not have a detrimental effect on BMI mediated by *Coprococcus* sp. According to the recommended dietary allowance (RDA), 1.1 - 1.2 mg of thiamin and 320–420 mg of magnesium intake are required to maintain normal physiological processes (41, 42). These RDAs values fall near the midpoint of the range of micronutrient intakes quantified from the dietary recall questionnaires of post-surgery patients (**Figures 3a, b**). Thus, patients whose intake exceeds the RDA may benefit from reducing their daily thiamin and magnesium intake to levels that are closer to the RDA. Future well-controlled studies to explore the feasibility of diet-driven modulation of the *Coprococcus* sp. abundance in order to enhance weight loss and augment the metabolic benefits of bariatric surgery are needed. In addition, a better understanding of thiamin consumption and/or production by the *Coprococcus* sp. is necessary as a study by Soto-Martin et al. (43) reported that not all *Coprococcus* spp. have either the presence of the genetic repertoire for *de novo* thiamin biosynthesis or show the same growth rate in thiamin-depleted medium. Isolation of *Coprococcus* sp. from post-bariatric surgery samples followed by *in vitro* and *in silico* analysis of its capacity to produce or use thiamin as well as whole-genome sequencing are necessary to clarify the identified positive thiamin-*Coprococcus* sp.-BMI association.

Interestingly, a negative relationship between *Coprococcus* sp. abundance and BMI was observed in obese patients before the bariatric surgery (**Figure 2d**). This is in contrast to the positive relationship found in post-surgery patients. Lozano et al. (44) provided insight into a possible mechanism underlying this opposite relationship. They reported a positive association between high inflammatory index diets and ectopic fat accumulation, but only in patients with lower gut *Coprococcus* abundance. Obese individuals typically consume high-inflammatory-index diets with high caloric content. Thus, obese patients in this study with lower *Coprococuss* sp. abundance may be more prone to accumulate ectopic fat and to have a higher BMI. Post-surgery patients follow less inflammatory diets which indicates that this mechanism does not affect them. However, other mechanisms may play a role as determinants of weight loss. For instance, *Coprococcuss* spp. are SCFA producers with the capacity to extract energy from otherwise undigested diet components (43). Therefore, in the altered post-surgical gut environment, greater *Coprococcus* sp. abundance combined with a less diverse microbiota and high metabolic efficiency for extracting energy from limited substrates would make it more difficult for patients to lose the obese phenotype. No correlation between thiamin/magnesium intake and *Coprococcus* sp. abundance was found in severely obese individuals.

The role of *Coprococcus* in health remains a subject of debate. While a large portion of the scientific literature associates this genus with beneficial traits such as improved insulin sensitivity in metabolically healthy obese individuals (7–9), reduced inflammation, enhance anti-inflammatory molecules, mucin production and tight junctions restoration (10, 11), other studies linked it to with small intestine bacterial overgrowth (12), depressive symptoms (13), inflammatory bowel disease (14), liver tumorigenesis (15) and interstitial cystitis (16). The gut microbiota is a diverse and complex community in which bacterial growth is tightly linked to the available resources. Thus, divergent associations with health and disease may result from differences in microenvironments, diseases and bacteria-bacteria interactions. The findings of this study should be interpreted in light of certain limitations. First, although false discovery rate adjustment was applied in multiple comparison hypotheses testing to control for false positives, replication in larger, independent cohorts from diverse geographical locations is needed to strengthen the conclusions. Second, major intestinal rearrangement following bariatric surgery has been reported as the main driver of most of the changes in the gut microbiota profile (45). However, the contribution of other confounders not evaluated in this study such as medication use and lifestyle modifications should not be excluded and warrants further investigation.

## 5 Conclusion

Important changes in clinical variables and microbiota were observed after individuals with severe obesity underwent malabsorptive bariatric surgery. Only 20% of patients who used hypoglycemic medication before surgery continued doing so afterwards, while the remaining 80% did not longer required medication to control their glucose levels. This improvement was accompanied by changes in the gut microbiota alpha and beta diversity. After surgery, the gut microbiota was less diverse, with a decrease in the abundance of several bacterial species. These changes in the microbiota profile led to a reduction in the enrichment of metabolic pathways involved in amino acid, thiamin, sugar degradation and acetate production. The abundance of a species of the genus *Coprococcus* showed opposite relationships with BMI in pre-surgery and post-surgery individuals. Furthermore, its abundance correlated with magnesium and thiamin intake in individuals who underwent a malabsorptive bariatric surgery.

## Data Availability

The datasets presented in this study can be found in online repositories. The names of the repository/repositories and accession number(s) can be found below: https://www.ncbi.nlm.nih.gov/, PRJNA1181870.
