# Supplementary material for: A species of Coprococcus is related to BMI in patients who underwent malabsorptive bariatric surgery and its abundance is modified by magnesium and thiamin intake
Source: Front Endocrinol (Lausanne). 2025 Nov 6;16:1613221. doi: 10.3389/fendo.2025.1613221 (PMC12631114; doi:10.3389/fendo.2025.1613221)
Supplement: Supplementary file 1 [file Table1.docx]

## Appendix A. Supplementary data

The following is the Supplementary data to this article:

Supplementary data 1.

Table S1: Quartiles and adjusted p values of phyla identified in patients before (labeled as 1) and after (labeled as 2) the bariatric surgery.

| **Phyla** | **P FDR** | **1 - Q25%** | **1 - Q50%** | **1 - Q75%** | **2 - Q25%** | **2 - Q50%** | **2 - Q75%** |
| --- | --- | --- | --- | --- | --- | --- | --- |
| Actinobacteria | 0.204 | 0.005 | 0.006 | 0.020 | 0.003 | 0.004 | 0.009 |
| Bacteroidetes | **0.014** | 0.170 | 0.323 | 0.427 | 0.423 | 0.517 | 0.601 |
| Firmicutes | **0.002** | 0.490 | 0.612 | 0.730 | 0.302 | 0.382 | 0.480 |
| Fusobacteria | **0.002** | 0.000 | 0.000 | 0.000 | 0.000 | 0.003 | 0.016 |
| Proteobacteria | 0.051 | 0.006 | 0.016 | 0.048 | 0.028 | 0.058 | 0.115 |
| Verrucomicrobia | 0.236 | 0.000 | 0.000 | 0.005 | 0.000 | 0.000 | 0.000 |
